# *Lactobacillus allii* sp. nov. isolated from scallion kimchi

**DOI:** 10.1099/ijsem.0.002327

**Published:** 2017-10-18

**Authors:** Min Young Jung, Se Hee Lee, Moeun Lee, Jung Hee Song, Ji Yoon Chang

**Affiliations:** Microbiology and Functionality Research Group, World Institute of Kimchi, Gwangju 61755, Republic of Korea

**Keywords:** *Lactobacillus allii*, kimchi, lactic acid bacteria, novel species

## Abstract

A novel strain of lactic acid bacteria, WiKim39^T^, was isolated from a scallion kimchi sample consisting of fermented chili peppers and vegetables. The isolate was a Gram-positive, rod-shaped, non-motile, catalase-negative and facultatively anaerobic lactic acid bacterium. Phylogenetic analysis of the 16S rRNA gene sequence showed that strain WiKim39^T^ belonged to the genus *Lactobacillus*, and shared 97.1–98.2 % pair-wise sequence similarities with related type strains, *Lactobacillus nodensis*, *Lactobacillus insicii*, *Lactobacillus versmoldensis*, *Lactobacillus tucceti* and *Lactobacillus furfuricola*. The G+C content of the strain based on its genome sequence was 35.3 mol%. The ANI values between WiKim39^T^ and the closest relatives were lower than 80 %. Based on the phenotypic, biochemical, and phylogenetic analyses, strain WiKim39^T^ represents a novel species of the genus *Lactobacillus*, for which the name *Lactobacillus allii* sp. nov. is proposed. The type strain is WiKim39^T^ (=KCTC 21077^T^=JCM 31938^T^).

Fermented foods and beverages not only provide important nutrients but also have great potential for maintaining health and preventing disease, and they play an important role in the human diet worldwide [1, 2]. Kimchi is the most well-known traditional fermented food in Korea, and it is made from various raw materials, such as napa cabbage, radish, red pepper, garlic, ginger, radish, and fermented seafood (jeotgal) [3]. Kimchi contains various vitamins and health-promoting components, and it provides health benefits such as antiobesity, anticancer, antioxidation, antimutagen, and anti-atherosclerotic effects [4–6]. In addition, many lactic acid bacteria (LAB) of the genera *Leuconostoc, Weissella* and *Lactobacillus* are involved in the fermentation process of kimchi [7]. The genus *Lactobacillus* belongs to the large group of LAB producing lactic acid by carbohydrate fermentation. These bacteria are characterized as Gram-positive, non-spore-forming rods, which have a low G+C content, and are catalase-negative, non-motile microorganisms [8, 9]. Members of the genus *Lactobacillus* are usually found in plants; plant-derived materials such as silage, grains, and foods; and the gastrointestinal tract of humans and animals. *Lactobacillus* strains are currently used as probiotics, starters, and silage inoculants in food and feed fermentation [10]. In the present study, a novel strain within the genus *Lactobacillus*, which is used in the food and feed industries, was isolated from scallion kimchi, and the phenotypic, chemotaxonomic, and molecular characteristics of the novel LAB strain WiKim39^T^ are presented.

Strain WiKim39^T^ was isolated from scallion kimchi in Gwangju, Korea, using the dilution plating method with MRS agar medium (MRSA; BBL) at 30 °C for 48 h. Single colonies on the plates were transferred to new plates and incubated on MRS agar at 30 °C under anaerobic conditions. The reference strains used in this study, *Lactobacillus nodensis* KACC 16346^T^, *Lactobacillus insicii* DSM 29801^T^, *Lactobacillus versmoldensis* KCTC 3814^T^, *Lactobacillus tucceti* KCTC 21005^T^ and *Lactobacillus furfuricola* KCTC 21034^T^, were cultured in MRS medium according to the culture collection guidelines. Cells of strain WiKim39^T^ grown on MRS were Gram-stained and visualized under light microscopy and transmission electron microscopy (TEM, Hitachi 7000 electron microscope). Bacterial growth at various pH values (3.0–10.0 in 0.5-unit increments) was measured by inoculating pH-adjusted MRS media with HCl or KOH. The optimum growth temperature and tolerance to NaCl of the cells were also measured. For these studies, cells were grown at temperatures ranging from 15 to 60 °C and in 0–10 % (w/v) NaCl for 48 h. Growth was monitored by measuring OD600 using a UV-1600 spectrometer (Shimadzu). Growth under anaerobic conditions was tested on MRS agar (Difco) using GasPak jars (BBL) at 30 °C. Motility was tested in MRS medium with 0.4 % agar. Physiological characteristics (acid production, carbon-source utilization, enzyme activities, and biochemical features) were determined using the API 50CH, API ZYM, API 20E, and API 20 Strep galleries according to the manufacturer’s instructions (bioMérieux). Catalase activity was determined by assessing the production of oxygen bubbles in 3 % (v/v) aqueous hydrogen peroxide solution. Oxidase activity was measured using an Oxy-swab (bioMérieux) according to the manufacturer’s recommendations. For haemolysis testing, the bacteria were streaked on MRS agar containing 5 % (w/v) sheep's blood and incubated for 48 h at 30 °C. Tellurite tolerance tests were performed by supplementation with 0.04 % K_2_TeO_3_ (Sigma-Aldrich). Lactic acid production was quantified using a dl-lactic acid assay kit (Megazyme International).

Cells of strain WiKim39^T^ were rods with an average length of 0.6×1.8–2.5 µm. The strain was identified as a facultative anaerobe, and it grew at temperatures from 25 to 37 °C and at a pH from 4.5 to 9.0 with optimum growth at 30 °C and pH 6.5–7.0. No growth was observed at ≤20 °C and ≤pH 4.0 or ≥40 °C and ≥7 % (w/v) NaCl l^−1^. Positive reactions were observed in tests for the production of acid from d-galactose, d-glucose, d-fructose, *N*-acetylglucosamine, amygdalin, arbutin, aesculin ferric citrate, salicin, d-cellobiose, d-maltose, d-lactose, d-sucrose, and gentiobiose. In addition, enzyme detection with an API zym kit was positive for alkaline phosphatase, esterase, leucine arylamidase, valine arylamidase, acid phosphatase, naphtol-AS-BI-phosphohydrolase, α-glucosidase, β-glucosidase, and *N*-acetyl-β-glucosaminidase. No esterase lipase, cystine arylamidase, trypsin, α-chymotrypsin, α-galactosidase, β-galactosidase, β-glucuronidase, α-mannosidase, or α-fucosidase activities were observed. Biochemical features were negative for nitrate and nitrite reduction; production of indole, H_2_S, urease, pyrrolidonyl arylamidase, arginine dihydrolase; or hydrolysis of gelatin and hippurate. WiKim39^T^ showed non-haemolysis (γ-haemolysis) and tellurite tolerance. The novel species could be differentiated from all other tested reference type strains using its physiological properties (Table 1). It was found to produce d- and l-lactic acid at a ratio of 52 : 48. Previously reported reference strains produce significantly more l-lactic acid than d-lactic acid, whereas strain WiKim39^T^ produced slightly more d-lactic acid than l-lactic acid [11–13].

**Table 1.** Differential characteristics of strain WiKim39^T^ compared with those of other species Strains: 1, *Lactobacillus allii* strain WiKim39^T^; 2, *Lactobacillus nodensis* KACC 16346^T^; 3, *Lactobacillus insicii* DSM 29801^T^; 4, *Lactobacillus versmoldensis* KCTC 3814^T^; 5, *Lactobacillus tucceti* KCTC 21005^T^; and 6, *Lactobacillus furfuricola* KCTC 21034^T^. All data are from this study. All strains were positive for d-glucose, d-fructose, *N*-acetylglucosamine, leucine arylamidase, valine arylamidase, β-glucosidase, and the Voges-Proskauer test. All strains were negative for indole, H_2_S, and urease production and hydrolysis of gelatin and hippurate. +, positive; −, negative.

**Characteristic**	**1**	**2**	**3**	**4**	**5**	**6**
Growth at/with						
15 °C	+	+	+	+	+	−
45 °C	−	−	+	−	−	−
10 % NaCl	−	−	+	+	+	+
pH 4.0	−	+	+	+	+	+
Acid production from:						
d-Arabinose	−	+	−	−	−	−
d-Ribose	−	+	+	+	+	+
d-Galactose	+	+	−	+	−	−
l-Rhamnose	−	−	−	−	+	−
d-Mannitol	−	−	−	−	+	−
Amygdalin	+	−	−	−	−	−
Arbutin	+	−	−	−	−	−
Aesculin ferric citrate	+	−	−	−	−	−
Salicin	+	−	−	−	−	−
d-Cellobiose	+	−	−	−	−	−
d-Maltose	+	−	+	+	+	+
d-Lactose	+	−	−	−	−	−
d-Melibiose	−	−	−	+	−	−
d-Sucrose	+	−	−	−	−	−
Gentiobiose	+	−	−	−	−	−
d-Turanose	−	−	+	−	−	−
l-Fucose	−	−	−	−	+	−
Enzyme activity						
Alkaline phosphatase	+	−	−	−	−	−
Esterase	+	−	−	−	−	−
Esterase lipase	−	−	−	−	−	+
Acid phospatase	+	+	+	−	−	+
Naphtol-AS-BI-phosphohydrolase	+	−	−	−	−	+
*α*-Galactosidase	−	−	−	+	−	−
β-Galactosidase	−	−	−	+	−	−
*α*-Glucosidase	+	+	−	+	−	−
β-Glucosidase	+	+	+	+	+	+
*N*-acetyl-β-glucosaminidase	+	−	−	−	−	−
Arginine dihydrolase	−	+	+	−	−	−
Pyrrolidonyl arylamidase	−	+	+	−	−	−
Citrate utilization	+	−	−	−	−	−
D: L lactate ratio*	52 : 48	4 : 96	10 : 90	13 : 87	17 : 83	30 : 70
DNA G+C content (mol%)*	35.3	40.6	36.3	38.3	34	40–40.8

*Data from: [11–14, 27, 28].

Bacterial DNA extraction was performed using the QIAcube system with a QIAamp DNeasy Blood and Tissue Kit (Qiagen). PCR amplification and sequencing of the 16S rRNA gene, RNA polymerase alpha subunit (*rpoA*), and phenylalanyl-tRNA synthase alpha subunit (*pheS*) gene were performed according to the method described by Irisawa *et al*. [14]. Full sequences of the 16S rRNA gene were compiled using SeqMan software (DNASTAR). The sequences of the 16S rRNA, *rpoA*, and *pheS* genes of the isolated strain were compared with available sequences from GenBank using the blast program (http://www.ncbi.nlm.nih.gov/blast/) to determine their approximate phylogenetic affiliation. 16S rRNA gene sequence similarities were determined using the EzTaxon-e server (http://www.ezbiocloud.net/) [15]. The phylogenetic analysis was performed using the software package mega (Molecular Evolutionary Genetics Analysis) version 7 [16] after multiple sequence alignment of the data using clustal_x [17]. Phylogenic trees were constructed using the neighbour-joining (NJ), minimum-evolution, and maximum-likelihood methods, and bootstrap values were calculated on the basis of 1000 replications.

Based on the pairwise 16S rRNA gene sequence similarities, the closest phylogenetic relatives to strain WiKim39^T^ were *Lactobacillus nodensis* DSM 19682^T^ (98.2 %), *Lactobacillus insicii* TMW1.2011^T^ (98.1 %), *Lactobacillus versmoldensis* KU-3^T^ (97.5 %), *Lactobacillus tucceti* CECT 5920^T^ (97.3 %), and *Lactobacillus furfuricola* JCM 18764^T^ (97.1 %). The 16S rRNA analysis clearly indicated that the isolate represented a novel genomic species in the genus *Lactobacillus*, as none of the valid described species showed more than 98.7 % 16S rRNA similarity [18]. The phylogenetic relationships between strain WiKim39^T^ and related *Lactobacillus* species are shown in Fig. 1.

**Fig. 1.**
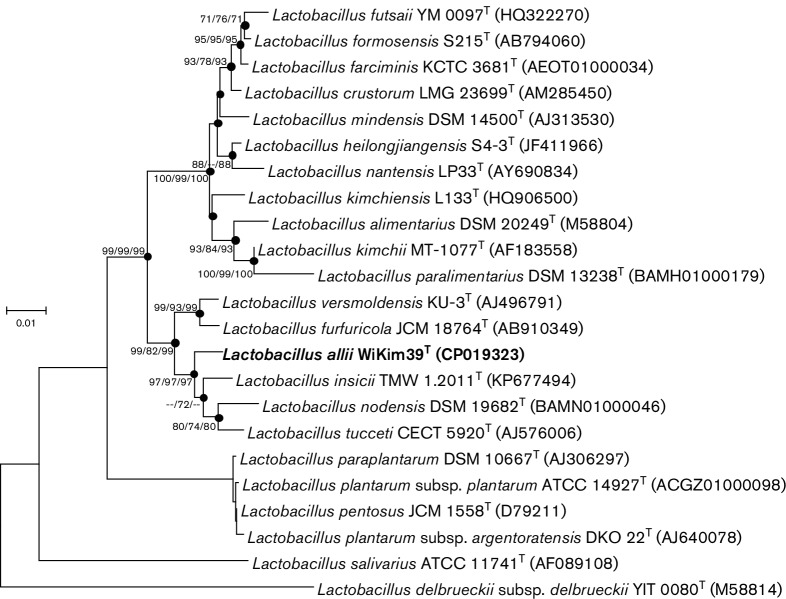
Neighbour-joining phylogenetic tree based on 16S rRNA gene sequences showing the relationships of strain WiKim39^T^ within the genus *Lactobacillus*. Filled circles represent identical branches that are present in phylogenetic consensus trees constructed using the neighbour-joining, maximum-parsimony, and minimum-evolution algorithms. Numbers at nodes indicate bootstrap values as percentages of 1000 replicates, and values >50 % are shown at branch points. *Lactobacillusdelbrueckii*subsp.*delbrueckii* YIT 0080^T^ was used as outgroup. Bar, 0.01 changes per nucleotide position.

Phylogenetic analysis based on the housekeeping genes encoding phenylalanyl-tRNA synthase alpha subunit (*pheS*) and RNA polymerase alpha subunit (*rpoA*) showed that the closest phylogenetic relative strains of strain WiKim39^T^ were type strains of *Lactobacillus nodensis*, *Lactobacillus insicii*, and *Lactobacillus tucceti*, with 84.4–87.6 % *pheS* gene sequence similarity and 89.5–94.5 % *rpoA* gene sequence similarity (Fig. 2). These gene sequence divergence values for *pheS* and *rpoA* between strain WiKim39^T^ and reference strains indicate that strain WiKim39^T^ represents a novel species within the genus *Lactobacillus* [19].

**Fig. 2.**
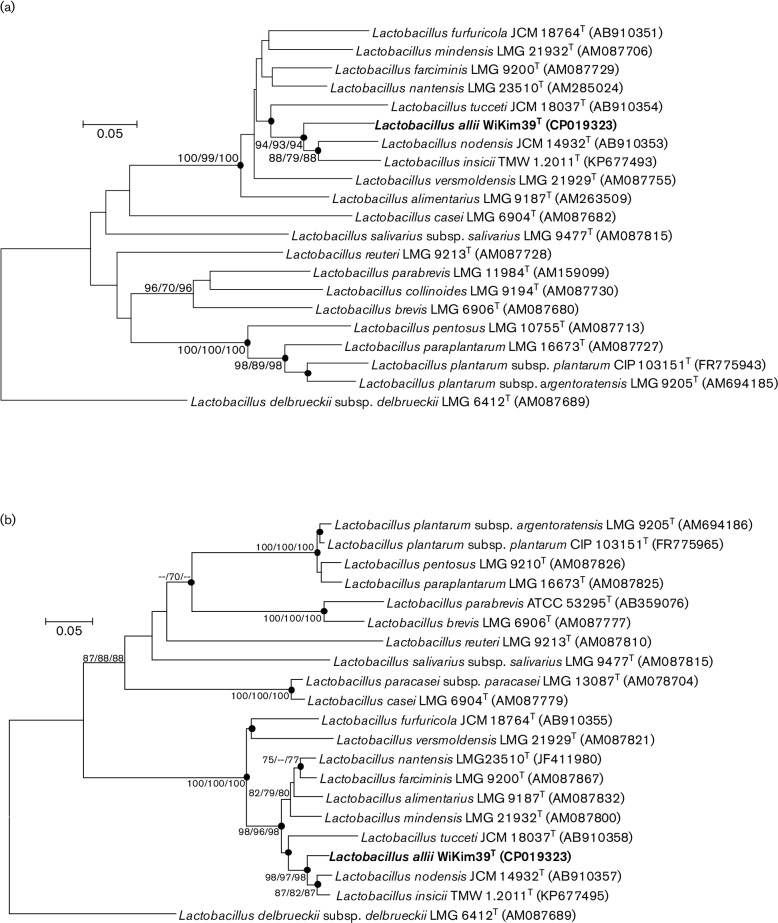
Neighbour-joining phylogenetic tree based on partial *pheS* (a) and *rpoA* (b) sequences showing the relationships between strain WiKim39^T^ and closely related species chosen as reference strains. Filled circles represent identical branches that are present in phylogenetic consensus trees constructed using the neighbour-joining, maximum-parsimony, and minimum-evolution algorithms. Numbers at nodes indicate bootstrap values as percentages of 1000 replicates, and values >50 % are shown at branch points. *Lactobacillusdelbrueckii*subsp.*delbrueckii* LMG 6412^T^ was used as outgroup. Bar, 0.05 changes per nucleotide position.

DNA–DNA hybridization was performed using the fluorometric hybridization method in microdilution wells as described previously [20]. The DNA–DNA hybridization values between strain WiKim39^T^ and *Lactobacillus nodensis* KACC 16346^T^, *Lactobacillus insicii* DSM 29801^T^, *Lactobacillus versmoldensis* KCTC 3814^T^, *Lactobacillus tucceti* KCTC 21005^T^, and *Lactobacillus furfuricola* KCTC 21034^T^ were 51.5, 48.1, 39.2, 29.3, and 30.7 %, respectively. The DNA–DNA relatedness values between the novel isolate and reference bacteria were significantly lower than the recommended threshold of 70 % for definition of a species [21].

The complete genome of strain WiKim39^T^ was sequenced using the PacBio RSII sequencing system (Pacific Biosciences) by Macrogen. The reads were assembled *de novo* using Hierarchical Genome Assembly Process version 3.0 (HGAP3.0) in SMRT analysis version 2.3.0 [22]. The complete genome sequence was annotated using the combined results from the automatic NCBI Prokaryotic Genomes Annotation Pipeline (PGAP).

The complete genome of strain WiKim39^T^ consists of a circular 2 506 167 bp chromosome and one circular plasmid, totaling 2 534 178 bp. The chromosome contains 2427 predicted protein-coding genes (CDCs), four rRNA operons (5S rRNA, 16S rRNA and 23S rRNA), 67 tRNAs, and 3 noncoding RNAs. Strain WiKim39^T^ contains 35.3 mol% G+C in its DNA, which is in the reported range of 31.93–57.02 % for *Lactobacillus* species [23], and this value was similar to the value of 37.6 % for *Lactobacillus nodensis* DSM 19682^T^ (AZDZ00000000). The complete genomes determined in this study have been deposited in the NCBI GenBank database under the accession numbers CP019323 (chromosome) and CP019324 (plasmid).

To evaluate the similarity between genome sequences, average nucleotide identity (ANI) values were analyzed between the strain and reported genomes using EzBioCloud as described by Moon *et al*. [24]. The strain showed ≤78.6 % ANI values relative to the *Lactobacillus* reference strains *Lactobacillus nodensis* DSM 19682^T^ (AZDZ00000000) and *Lactobacillus tucceti* DSM 10183^T^ (AZDG01000001), and the strain was therefore concluded to represent a novel species [25]. These DNA relatedness, DNA–DNA hybridization, and ANI results indicate that strain WiKim39^T^ represents a novel genomic species that is distinct from its closest relatives.

The cell-wall peptidoglycan type was determined by methods described previously [26]. Cellular fatty acid patterns were determined in cells from all reference strains and strain WiKim39^T^ grown on MRSA plates at 30 °C for 48 h. The fatty acid methyl esters were extracted and analyzed according to the standard protocol of the Sherlock Microbial Identification System (MIS, MIDI Inc.). The fatty acid methyl ester mixtures were separated with an automated GC system (autosampler models 7890A and 7683B; Agilent) and identified using the BHIBLA database of the Microbial Identification Sherlock software package v6.3.

The peptidoglycan structure type of strain WiKim39^T^ was A4*α* l-Lys-d-Asp (A11.31), which is the major type in most species of the closely related reference strains and the *L. alimentarius – L. farciminis* phylogenetic subgroup within the genus *Lactobacillus* [27]. The major cellular fatty acids contained by WiKim39^T^ were C_16 : 0_ and C_18 : 1_ω9*c*. The fatty acid composition of WiKim39^T^ was similar to that of *L. tucceti* KCTC 21005^T^, with small variations in the proportion, whereas the major fatty acids of the other reference strains were C_16 : 0_, C_18 : 1_ω9*c*, and C_19 : 1_ cyclo 9, 10 (Table 2). On the basis of its phenotypic, genotypic, and chemotaxonomic characteristics, strain WiKim39^T^ can thus be distinguished clearly from the type strains of other species of the genus *Lactobacillus*, and the name *Lactobacillus allii* sp. nov. is proposed.

**Table 2.** Cellular fatty acid compositions of strain WiKim39^T^ and related *Lactobacillus* species Strains: 1, *Lactobacillus allii* strain WiKim39^T^; 2, *Lactobacillus nodensis* KACC 16346^T^; 3, *Lactobacillus insicii* DSM 29801^T^; 4, *Lactobacillus versmoldensis* KCTC 3814^T^; 5, *Lactobacillus tucceti* KCTC 21005^T^; and 6, *Lactobacillus furfuricola* KCTC 21034^T^. Values are percentages of total fatty acids. tr, trace amount (0.5–1.0 %); –, not detected.

**Fatty acid**	**1**	**2**	**3**	**4**	**5**	**6**
C_10 : 0_	tr	tr	tr	1.2	tr	1.4
C_16 : 1_ω*9c*	1.2	1.1	1.1	1.1	1.1	1.2
C_16 : 0_	16.8	17.2	16.9	22.7	17.6	23.4
C_18 : 1_ω*9c*	61.9	27.1	40.8	21.8	62.5	34.9
C_18 : 0_	2.4	3.8	2.8	5.7	2.3	3.9
C_18 : 1_ω1*1c* DMA	1.5	1.6	2.5	2.3	1.7	3.1
Un (ECL 18.199) C_18 : 0 a_DMA	2.2	1.8	2.9	2.5	2.3	2.5
C_19_ cyc 9, 10/:1 FAME	1.1	33.6	20.4	26.8	–	15.9
Summed feature 10*	8.1	8.8	8.3	10.3	7.6	9.7
Summed feature 12*	2.4	2.7	2.6	3.8	3.3	2.5

*Fatty acids that could not be separated by GC using the microbial identification system (Microbial ID) software were considered summed features. Summed feature 10 contains one or more of an unknown fatty acid of C_18 : 1_ω1*1c*/9 t/6 t and/or ECL 17.834. Summed feature 12 contains one or more of an unknown fatty acid of ECL 18.622 and/or iso-C_19 : 0_.

## Description of *Lactobacillus allii* sp. nov.

*Lactobacillus allii* (al′li.i. L. gen. n. *allii* of garlic, of the botanical genus *Allium*, the source of scallion kimchi from which the type strain was isolated) cells are Gram-positive, catalase and oxidase-negative, facultatively anaerobic, and non-motile. Additionally, the cells are non-spore-forming rods, are 0.6×1.8–2.5 µm in size, and occur singly or in pairs. Colonies grown on MRS agar at 30 °C for 48 h are up to 1.0 mm in diameter, off-white, smooth, and round with rough surfaces. They are homofermentative; gas is not produced from glucose. The cells produce d- and l-lactic acid at a ratio of 52 : 48. Growth occurs at 25–37 °C and in the presence of 5 % NaCl but not in the presence of 7 % NaCl. Growth occurs at pH 4.5–9.0 but not at pH 3.5–4.0. Acid is produced from d-galactose, d-glucose, d-fructose, *N*-acetylglucosamine, amygdalin, arbutin, aesculin ferric citrate, salicin, d-cellobiose, d-maltose, d-lactose, d-sucrose, and gentiobiose. Acid is not produced from glycerol, erythritol, arabinose, ribose, xylose, d- or l-arabinose, adonitol, methyl β-d-xylopyranoside, sorbose, rhamnose, dulcitol, inositol, mannitol, sorbitol, methyl α-d-mannopyranoside, methyl α-d-glucopyranoside, melibiose, trehalose, inulin, melezitose, raffinose, starch, glycogen, xylitol, turanose, d-lyxose, d-tagatose, d- or l-fucose, d- or l-arabitol, potassium gluconate, potassium 2-ketogluconate, or potassium 5-ketogluconate. Alkaline phosphatase, estalase, leucine arylamidase, valine arylamidase, acid phosphatase, naphtol phosphohydrolase, α-glucosidase (maltase), β-glucosidase (cellulose), and *N*-acetyl-β-glucosaminidase (chitinase) are produced. Nitrate is not reduced. Indole and H_2_S gas are not produced. The Voges-Proskauer test is positive. Tellurite tolerance is present. The cell-wall peptidoglycan structure type is A4α l-Lys–d-Asp. The major cellular fatty acids are C_16 : 0_ and C_18 : 1_ω9*c*. The DNA G+C content of the type strain is 35.3 mol%, and the genome size is 2.53 Mb. The type strain WiKim39^T^ (=KCTC 21077^T^=JCM 31938^T^) was isolated from scallion kimchi in Gwangju, Korea.

## References

[1] Kabak B, Dobson AD (2011). An introduction to the traditional fermented foods and beverages of Turkey. Crit Rev Food Sci Nutr.

[2] Rolle R, Satin M (2002). Basic requirements for the transfer of fermentation technologies to developing countries. Int J Food Microbiol.

[3] Kim M, Chun J (2005). Bacterial community structure in kimchi, a Korean fermented vegetable food, as revealed by 16S rRNA gene analysis. Int J Food Microbiol.

[4] Patra JK, Das G, Paramithiotis S, Shin HS (2016). Kimchi and other widely consumed traditional fermented foods of Korea: a review. Front Microbiol.

[5] Lee G-I, Lee H-M, Lee C-H (2012). Food safety issues in industrialization of traditional Korean foods. Food Control.

[6] Park KY, Jeong JK, Lee YE, Daily JW (2014). Health benefits of kimchi (Korean fermented vegetables) as a probiotic food. J Med Food.

[7] Jung JY, Lee SH, Jeon CO (2014). Kimchi microflora: history, current status, and perspectives for industrial kimchi production. Appl Microbiol Biotechnol.

[8] Swain MR, Anandharaj M, Ray RC, Parveen Rani R (2014). Fermented fruits and vegetables of Asia: a potential source of probiotics. Biotechnol Res Int.

[9] Hammes WP, Weiss N, Holzapfel W, Balows A, Trüper HG, Dworkin M, Harder W, K-H Schleifer (1991). The genera *Lactobacillus* and *Carnobacterium*. In The Prokaryotes: a Handbook on the Biology of Bacteria: Ecophysiology, Isolation, Identification, Applications.

[10] Giraffa G, Chanishvili N, Widyastuti Y (2010). Importance of lactobacilli in food and feed biotechnology. Res Microbiol.

[11] Ehrmann MA, Kröckel L, Lick S, Radmann P, Bantleon A (2016). *Lactobacillus insicii* sp. nov., isolated from fermented raw meat. Int J Syst Evol Microbiol.

[12] Kröckel L, Schillinger U, Franz CM, Bantleon A, Ludwig W (2003). *Lactobacillus versmoldensis* sp. nov., isolated from raw fermented sausage. Int J Syst Evol Microbiol.

[13] Chenoll E, Carmen Macián M, Aznar R (2006). *Lactobacillus tucceti* sp. nov., a new lactic acid bacterium isolated from sausage. Syst Appl Microbiol.

[14] Irisawa T, Tanaka N, Kitahara M, Sakamoto M, Ohkuma M (2014). *Lactobacillus furfuricola* sp. nov., isolated from Nukadoko, rice bran paste for Japanese pickles. Int J Syst Evol Microbiol.

[15] Kim OS, Cho YJ, Lee K, Yoon SH, Kim M (2012). Introducing EzTaxon-e: a prokaryotic 16S rRNA gene sequence database with phylotypes that represent uncultured species. Int J Syst Evol Microbiol.

[16] Kumar S, Stecher G, Tamura K (2016). MEGA7: molecular evolutionary genetics analysis version 7.0 for bigger datasets. Mol Biol Evol.

[17] Thompson JD, Gibson TJ, Plewniak F, Jeanmougin F, Higgins DG (1997). The CLUSTAL_X windows interface: flexible strategies for multiple sequence alignment aided by quality analysis tools. Nucleic Acids Res.

[18] Stackebrandt E, Ebers J (2006). Taxonomic parameters revisited: tarnished gold standards. Microbiol Today.

[19] Naser SM, Dawyndt P, Hoste B, Gevers D, Vandemeulebroecke K (2007). Identification of lactobacilli by *pheS* and *rpoA* gene sequence analyses. Int J Syst Evol Microbiol.

[20] Ezaki T, Hashimoto Y, Yabuuchi E (1989). Fluorometric deoxyribonucleic acid-deoxyribonucleic acid hybridization in microdilution wells as an alternative to membrane filter hybridization in which radioisotopes are used to determine genetic relatedness among bacterial strains. Int J Syst Bacteriol.

[21] Wayne LG (1988). International committee on systematic bacteriology: announcement of the report of the ad hoc committee on reconciliation of approaches to bacterial systematics. Zentralbl Bakteriol Mikrobiol Hyg.

[22] Chin CS, Alexander DH, Marks P, Klammer AA, Drake J (2013). Nonhybrid, finished microbial genome assemblies from long-read SMRT sequencing data. Nat Methods.

[23] Sun Z, Harris HM, McCann A, Guo C, Argimón S (2015). Expanding the biotechnology potential of lactobacilli through comparative genomics of 213 strains and associated genera. Nat Commun.

[24] Moon JS, Choi HS, Shin SY, Noh SJ, Jeon CO (2015). Genome sequence analysis of potential probiotic strain *Leuconostoc lactis* EFEL005 isolated from kimchi. J Microbiol.

[25] Ramasamy D, Mishra AK, Lagier JC, Padhmanabhan R, Rossi M (2014). A polyphasic strategy incorporating genomic data for the taxonomic description of novel bacterial species. Int J Syst Evol Microbiol.

[26] Schumann P (2011). Peptidoglycan structure. Methods Microbiol.

[27] Kashiwagi T, Suzuki T, Kamakura T (2009). *Lactobacillus nodensis* sp. nov., isolated from rice bran. Int J Syst Evol Microbiol.

[28] Kim DS, Choi SH, Kim DW, Kim RN, Nam SH (2011). Genome sequence of *Lactobacillus versmoldensis* KCTC 3814. J Bacteriol.

